# Human Stem Cells for Ophthalmology: Recent Advances in Diagnostic Image Analysis and Computational Modelling

**DOI:** 10.1007/s40778-023-00229-0

**Published:** 2023-11-18

**Authors:** L. E. Wadkin, I. Makarenko, N. G. Parker, A. Shukurov, F. C. Figueiredo, M. Lako

**Affiliations:** 1 School of Mathematics, Statistics and Physics, Newcastle University, Newcastle upon Tyne, UK; 2 Department of Ophthalmology, Royal Victoria Infirmary, Newcastle upon Tyne Hospitals NHS Foundation Trust, Newcastle upon Tyne, UK; 3 Biosciences Institute, Faculty of Medical Sciences, Newcastle University, Newcastle upon Tyne, UK

**Keywords:** Ophthalmology, Diagnostic image analysis, Mathematical modelling, Human stem cells, Machine learning, Agent-based modelling

## Abstract

**Purpose of Review:**

To explore the advances and future research directions in image analysis and computational modelling of human stem cells (hSCs) for ophthalmological applications.

**Recent Findings:**

hSCs hold great potential in ocular regenerative medicine due to their application in cell-based therapies and in disease modelling and drug discovery using state-of-the-art 2D and 3D organoid models. However, a deeper characterisation of their complex, multi-scale properties is required to optimise their translation to clinical practice. Image analysis combined with computational modelling is a powerful tool to explore mechanisms of hSC behaviour and aid clinical diagnosis and therapy.

**Summary:**

Many computational models draw on a variety of techniques, often blending continuum and discrete approaches, and have been used to describe cell differentiation and self-organisation. Machine learning tools are having a significant impact in model development and improving image classification processes for clinical diagnosis and treatment and will be the focus of much future research.

## Introduction

The ability of human stem cells (hSCs) to self-renew and differentiate into other human body cell types puts them at the forefront of progress in cellular therapies, disease modelling, and drug discovery. An area of regenerative medicine in which hSC research is particularly driving the development of translational research is ophthalmology.

Damage to the eye can be caused by disease or trauma and has a major impact on quality of life. The leading causes of vision impairment are cataracts, age-related macular degeneration (AMD), glaucoma, diabetic retinopathy, cornea opacity, and trachoma [1]. Injuries can also cause eye damage and vision loss, through mechanical, chemical, and thermal burns or radiation.

Stem cell-based therapies are being explored to treat a wide range of these debilitating eye conditions [2]. For example, pioneering limbal stem cell (LSC) therapies have been established to restore the sight of patients with unilateral LSC deficiencies, involving transplantation of ex vivo cultured LSCs taken from the contra-lateral healthy eye of the patient [3, 4].

The development of human pluripotent stem cell (hPSC)-derived organoids of ocular structures, including the cornea, retina, and lens [5–12], has also proved to be a transformative technology. These in vitro self-organising 3D structures which mimic the properties of their in vivo counterparts have become frequently utilised as models of human organ development [13, 14] and disease [12], to validate gene therapies [15, 16], and have the potential to provide a source of cells for cell-based therapy and transplantation [17, 18].

Mathematical and statistical modelling is an indispensable tool in supporting these advancements, deepening our knowledge of the fundamental mechanisms of hSC behaviour and providing descriptive and quantitative tools for in silico experimentation [19–21]. Increasingly, machine learning (ML) techniques are being used to assist with in vitro image analysis and in vivo image classification as an aid to clinical diagnosis and to assess response to treatment and prediction, control, and optimisation of differentiation pathways [22, 23].

In this review paper, we provide an overview of the recent advancements in the computational, mathematical, and statistical modelling of hSCs, with particular focus on applications in regenerative medicine within ophthalmology. We consider image analysis and image classification for diagnostic testing, the prediction of differentiation pathways, the modelling of 3D organoid structures, and the role of parameter inference in model development. Finally, we conclude and outline directions for future research in the field.

## Image Analysis and Classification

Computational techniques are a powerful tool to quantify, characterise, compare, and classify both in vitro and in vivo cellular images. In the in vitro case, this is essential for deepening our understanding of the fundamental properties of the cellular system and optimising in vitro experiments. In the in vivo case, computational techniques can be harnessed for the automatic extraction of clinically relevant information, such as identifying and evaluating diagnostic criteria, grading disease severity, and monitoring disease progression and the response to treatment.

For example, in limbal stem cell deficiency (LSCD) and stem cell therapies, the differences between healthy, diseased, and pre- and post-operative corneal cellular microscopy are often subtle and not easily discernible and clinical manifestations of different corneal diseases may appear similar. Thus, automated, quantitative tools capable of identifying objectively individual and collective cell features (and their differences between different cell populations) which are difficult or impossible to discern by slit lamp biomicroscopy are new technology that will have a significant impact on disease diagnostics and the monitoring of disease progression and response to treatment [24]. Previous analysis of in vivo confocal microscopy (IVCM) images of the cornea from patients with LSCD before and after LSC transplantation allowed the quantification of the size and density of corneal and conjunctival epithelial cell populations, uncovering statistically significant morphological differences between normal corneal cells and conjunctival epithelial cells and suggesting cell size and density (i.e. the average number of cells per unit area) not only as possible diagnostic measures of LSCD but also as an accurate measure of the surgical success [25]. Recent developments focus on automating such analysis of LSC images, for example, the development of an algorithmic ImageJ software package to automatically detect cell locations and provide cell density estimations [26].

**Fig.1:**
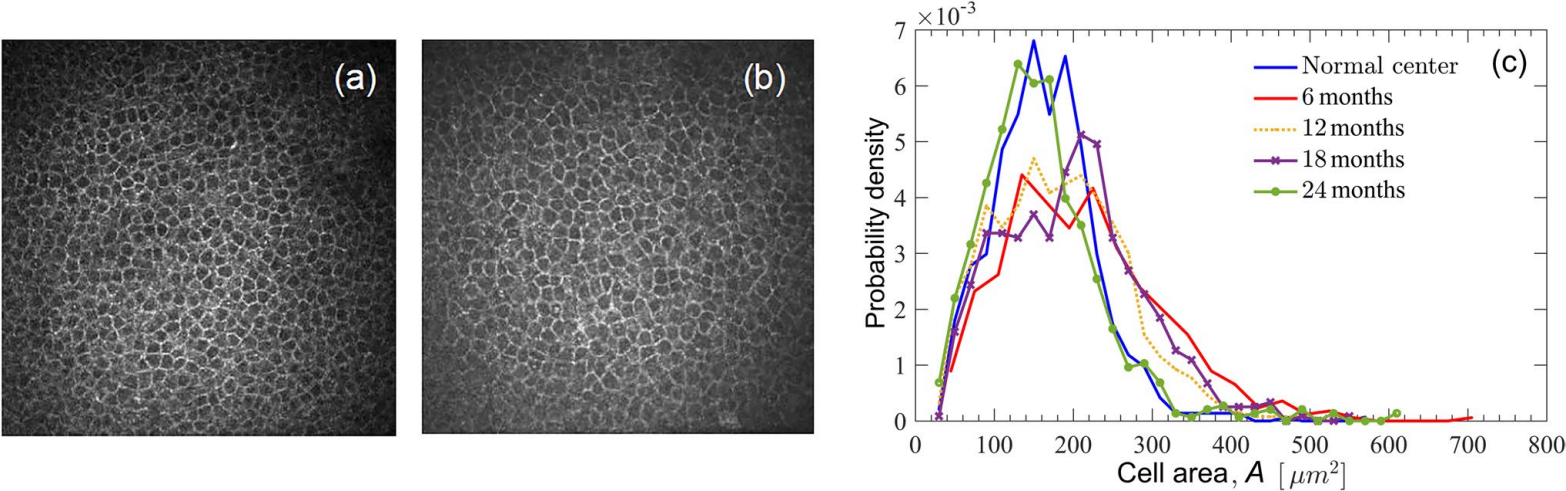
**a** An IVCM image of a healthy cornea epithelium (5 µm depth). **b** Similar image of the previously damaged cornea in the other eye of the same patient (same depth) 6 months after LSC transplantation. The difference from the healthy cornea is subtle and not easily discernible. **c** The probability density function of the cell sizes in the image of panel **a** (blue) and the post-operative cornea at 6 (as in panel **b**, red), 12, 18, and 24 months after surgery (as specified in the legend). The probability density at 6–18 months after the operation is clearly abnormal but recovers 24 months after the surgery (green line)

The results of computer-based image analysis often confirm and quantify diagnostic criteria known to the clinical practitioners. Even then, the quantification provided by image analysis techniques provides opportunities for a refinement of such criteria and a more objective assessment and diagnosis. On the other hand, some diagnostic features believed to be a useful discriminator of healthy and damaged cell populations turn out to be unreliable when assessed quantitatively. As an example of the former, the average size and density of cornea epithelial cells can be used for diagnosis and post-operative monitoring of the LSC deficiency and regeneration [25]. Moreover, the probability distribution of the cell sizes (i.e. the fraction of cells of any given size in the population) has been shown to be an even more sensitive discriminator of the state of the cell population (see Fig. 1) [27]. On the other hand, the well-known hexagonal shape (in other words, that each cell has on average six contact neighbour cells) was not confirmed to be a reliable signature of a healthy cornea epithelium [27].

Even more importantly, quantitative methods, especially combined with mathematical modelling, will lead to the discovery of new diagnostic features which are not discernible under visual image inspection. Such methods often require new approaches to the quantification and statistical comparison of images. Of particular importance is the need to identify (often subtle) diagnostic features of healthy and damaged cell populations through a comparison of many images. A promising and rapidly developing tool for this is machine learning (ML), an algorithmic approach trained on data without explicit instruction, and particularly, deep learning [28], a type of machine learning which uses a multi-layered neural network to automatically extract features from an image, allows a more sophisticated approach to cellular image analysis problems [29].

ML tools have the potential to aid clinical diagnosis. For example, a deep learning technique has been employed in a pilot study to diagnose and classify the severity grading of LSCD from IVCM images [30••]. A convolutional neural network (CNN) was trained first to identify representative scans of central basal cells and sub-basal nerves and then to classify LSCD severity in each case. Incorporating both cell and nerve IVCM images into the model resulted in more accuracy upon testing (74%) compared to cell scans only (68%) and nerve scans only (69%) [30••]. Although this approach shows promise for diagnostic utility, the methods do not directly quantify diagnostic markers of LSCD (e.g. epithelial thickness, cell morphology, density) which may be desirable for clinical interpretability [30••].

The diagnostic potential of ML techniques in ophthalmology is broad, with current research exploring the diagnosis of neuropathic corneal pain from IVCM images [31], the classification of diabetic peripheral neuropathy from corneal IVCM images [32], the detection of diabetic retinopathy from colour retinal fundus images [33], and multi-disease models to detect a range of common corneal diseases (dry eye syndrome, Fuchs endothelial dystrophy, and keratoconus) [34].

ML approaches can also be employed to further our understanding of the inherent properties of a cellular system. For example, a deep learning approach was developed and implemented to generate a complete morphometric retinal pigment epithelium (RPE) map of the human eye [35••]. The deep learning software (REShAPE: Retinal Epithelium Shape and Pigment Evaluator) helps to recognise and analyse RPE cell borders from flatmount images, leading to the identification of five statistically different RPE subpopulations (with different susceptibilities to aging and different types of retinal degenerative disease) using cell area. REShAPE uses a trained CNN to segment the images (i.e. to outline the cell boundaries) and then extract geometrical parameters (e.g. area, perimeter, shape, orientation) and the number of neighbours. The results of this RPE segmentation again point to cell area as a useful diagnostic metric, confirming earlier results based on other computational tools such as the CellProfiler image processing software [36]. The morphometry was also compared to hPSC-derived RPE cells to assess the variability of the subpopulation properties. Such software has the potential to be further expanded to incorporate the extraction of intra-cellular properties, such as genomic and transcriptomic markers through fluorescent imaging.

Similar ML tools can be applied to the analysis of microscopy images of in vitro cell cultures to deepen our understanding of their fundamental behaviours, for example, the quantitative description of migration and motility, key stem cell properties shown to impact differentiation pathways [37]. Research in this area traditionally relied on manual or semi-automated computer-based approaches to extract movement data from in vitro microscopy images [38, 39]. Such semi-automated methods often rely on the segmentation of the images (and other complex image pre-conditioning techniques) to identify an individual cell as it moves. However, now the deep learning neural network approach can be adapted to rely on the identification of a target cell in the beginning frame alone [40•]. In this case, a transfer learning method (in which the pre-trained model is reused) by the neural network facilitates the learning of invariant robust features of hPSCs for both tracking and mitosis detection [40•].

It is clear that ML approaches are driving significant developments in the quantification of in vitro stem cell properties and in in vivo image classification for diagnostic criteria, but their ‘black box’ nature (e.g. the lack of transparency in the reasons for a conclusion suggested by the software) and the substantial amounts of training data required can pose a challenge [29]. The current increasing interest and investment in obtaining, optimising, and managing efficient ‘big data’ for medical imaging will be beneficial in the expansion of ML applications [29].

## Prediction of Differentiation Pathways

A deeper understanding of pluripotency regulation and the prediction of differentiation pathways is required for the control and optimisation of ex vivo differentiation trajectories for the generation of retinal [41–44] and corneal [45–47] epithelial lineages and ocular organoids [5–9, 11, 14, 48], as well as for furthering knowledge of human development [49].

Many mathematical and statistical models have been explored for describing various aspects of differentiation behaviours, including proposed generalised differentiation pathways [50, 51], the regulation of intra-cellular pluripotency transcription factors [19, 52–56], the impact of cell-cell interactions [57], and the colony-scale spatial patterning in cell differentiation [57–60]. Previous models have also explored a computational approach to capturing the gene regulatory network (GRN), including a network model which describes 17 pluripotency factors to derive a set of components and interaction combinations to explain observed embryonic stem cell behaviour [61], and a Boolean simulation framework to predict signal-controlled GRN logic and stem cell fate decisions [62]. Similar models have recently been incorporated into multi-scale models which also describe cell size, position, and proliferation, as well as the GRN, allowing the exploration of the effects of the GRN on tissue patterning, composition, and dynamics [63].

Since differentiation is highly variable and influenced by a wide range of intra- and extra-cellular factors, many of which are hidden or unknown, pluripotency is often described using stochastic models, which account for the inherent random variations in the system, such as stochastic differential equations (SDEs) and stochastic network models. Recent SDE models include the stochastic logistic equation for the fluctuation of the pluripotency factor Oct4 in hPCs [19] and a model based on the stochastic Fokker-Planck equation model for cell state transitions in induced pluripotent stem cells (iPSCs) [64•]. Stochastic network models have been used to describe the differentiation of mouse embryonic cells [65•] and mapping the epigenetic Oct4 gene regulatory network [66]. Further details on the methodology of stochastic processes and their applications in cellular biology can be found in Refs. [67, 68].

It has been suggested that it is more useful to think of pluripotency as a statistical property (macro-state) of a stem cell population as a whole, as pluripotency is not well defined at the single-cell level [69], leading to models of cell fate decisions which use methodologies from statistical mechanics [70]. For example, analysis of mouse embryonic stem cell differentiation through the neuronal lineage found cell transition through a chain of unobserved molecular states in a stochastic, sequential manner, leading to a description of cell differentiation as a non-Markovian stochastic process (as opposed to a Markovian process, in which such transitions depend on some previous states, which is described as a ‘memory effect’) [71]. A recent review of the relevant concepts of statistical mechanics, including entropy, stochastic processes, and critical phenomena, in relation to single-cell biology can be found in Ref. [49].

Developments in ML are also facilitating advancements in the mathematical descriptions of pluripotency. CNNs have been used to assess the multipotency level of human nasal turbinate stem cells from several donors based on cell morphology in confocal images with 85% accuracy when compared to differentiation efficacy via in vitro and ex vivo assessments [72••]. They have also been used to predict differentiation in retinal organoids based on bright-field imaging, before the onset of reporter gene expression [73••]. Upon comparison with a human-based classifier, the neural network algorithm performed better than an expert in predicting organoid fate (84% vs 67%), demonstrating the power of neural networks for predicting differentiation in retinal organoids.

CNNs have also been applied to the classification of iPSC-derived cells to evaluate the differentiation efficiency of iPSC retinal pigmental epithelium cells [74••]. The model showed capabilities to classify iPSCs, iPSC-RPEs, and iPSC-retinal ganglion cells with an accuracy of 97.8% and accurately recognised the differentiation of iPSC-RPEs, suggesting that such a rapid screening/classification system could facilitate the translation of iPSC-based technologies into clinical uses. Another CNN has been able to identify vascular endothelial cells derived from iPSCs without the need for immunostaining or lineage tracing, based purely on cell morphology from phase contrast images [75].

The understanding of the cell differentiation pathways has developed to involve more and more sophisticated mathematical concepts and models. As in other applications of mathematical tools in biology, close collaboration between mathematicians and biologists, including laboratory experiments targeted to verify and refine theoretical models, is essential for further progress.

## 3D Modelling of Organoids

The development of ocular organoids derived from hPSCs has been a crucial advancement in stem cell science, providing a comprehensive and realistic in vitro model reproducing the fundamental properties of its in vivo counterpart and facilitating the study of human development and disease. Corresponding three-dimensional (3D) computational models can provide a complementary in silico means of experimentation and prediction.

Computational models for organoids vary in complexity, detail, scale, and the fundamental properties they seek to describe. Many models take a mechanistic biophysical approach, seeking to describe the cellular self-organisation, mechanical forces, and molecular signalling centred on known physical and biochemical principles [76–78]. Some models are continuum-based, focusing on approximating the biophysical properties on a macro-scale level using differential equations (i.e. describing an organoid as a whole rather than as a population of individual cells). Continuum models are well suited to describing overall mechanical behaviours of tissues [79], including self-organisation [60, 80]. A recent continuum model has been used to explore the optimisation of organoid culturing in a bioreactor, describing the spatio-temporal transport of key metabolites in terms of a system of partial differential equations [81•].

An alternative approach is discrete modelling (e.g. agent-based models (ABMs), individual based models, cellular automaton models), encompassing both lattice and lattice-free models, where the behaviour of each individual cell or group of cells is described. Discrete models are a powerful tool for modelling 3D cellular structures, including ocular organoids, as they reveal how the interactions of individual cells (micro-scale features) affect the large-scale processes (macro-scale behaviour). Such models are well suited for exploring spatial patterning and self-organisation.

ABMs have been suggested as a valuable technique for modelling retinal cell transplantation, due to their ability to capture a specific spatial architecture (e.g. the columnar structure of photoreceptor cells), incorporate heterogeneity (e.g. including a range of cell types with different mechanical, migratory, and chemical response behaviours), and include the intrinsic stochasticity [82]. Various forms of discrete models have also been shown to be capable of capturing collective properties that appear in organoids, for example, collective cell migration [83].

Combined with the clonal analysis, an ABM was used to show that retinal stem cells modulate the proliferative parameters that coordinate post-embryonic morphogenesis in the eye of fish [84•]. This model, developed in an earlier work [85], allows the spherical cells to move freely off-lattice, equilibrating their distance to neighbours by exerting pressure or adhesion forces, with the movements constrained to the hemispherical surface area of a growing eye globe. Cell division occurs probabilistically until the local cell density becomes too high, with the daughter cells positioned to initially overlap but gradually separating by displacing their neighbour cells. The model is used to investigate two synthetic growth mechanisms (‘inducer’ growth where cell division drives the growth of the eye radius and ‘responder’ growth where the growth of the eye globe radius stimulates cell division). The simulations showed that the inducer and responder growth modes differ in the variability of the cell division timing, resulting in distinct clonal patterns that reproduced experimentally observed differences between neural retina (described by ‘inducer’ growth) and RPE (described by ‘responder’ growth) [84•]. An overview of a range of mechanical models for retinal organoid morphological organisation can be found in Ref. [86].

ABMs have also been used to explore the mechanisms of corneal wound healing following chemical exposure [87]. Using the open-access CompuCell3D software [88], the ABM describes the differentiation and proliferation of several epithelial cell types throughout the regeneration process of the corneal epithelium under homeostasis and varying levels of injury severity [87]. The model has the corneal epithelial structure as an emergent feature and recapitulates the time of recovery for slight and mild injuries [87] but deviates from expected in vivo recovery for moderate injuries due to the lack of necessary cell types (e.g. keratocytes) required for full restoration. The next step in model development is to integrate bioactivity data from in vitro models of corneal toxicity and predict human-relevant adverse outcomes, including loss of structural integrity, time to recovery, and area and density of opacification [87].

Modelling techniques can also be combined to identify the key cell and environment properties of these complex multi-scale systems. For example, a model for the self-organisation of hPSC-derived optic-cup organoids takes an agent-based approach to the cellular structure, using a 3D vertex model, coupled with a continuum approach to the inter-cellular biochemical signalling based on a reaction-diffusion mechanism [89]. This model enabled a quantitative prediction of morphogenesis and suggests that mechanical forces play a key role as a feedback regulator in self-organising the 3D optic-cup formation. Such a synthetic modelling approach deserves further development.

It is not surprising that ML is also becoming influential in 3D organoid modelling through its ability to provide insights in differentiation pathways and colony organisation [90, 91] and enhance the efficiency of 3D cell-detection software [92]. Recent synergies between ML and ABMs have the potential to advance the development of biomedical system models even further, with ML used as a tool to infer optimal, application-specific ABM rules, while ABM simulations provide means to generate large amounts of data, which can then be fed into an ML framework [93]. A review of ML methods in organoid modelling can be found in Ref. [94].

## Model Parameter Inference

Maximising the predictive power of any mathematical or computational model of a biological system requires the inference of representative parameters from both the real (e.g. from a laboratory experiment) and modelled data. Furthermore, adequate statistical tests must be applied to assess the level of agreement between different parameter measurements, either experimental or resulting from modelling. However, it is often difficult or impossible to obtain experimental data required to understand the biophysical properties of each element of a complex, multi-scale model. There are also common data quality issues such as incompleteness and biases. Furthermore, the inherent stochasticity and multi-scale complexity of many of these models can make the implementation of sophisticated statistical inference techniques computationally challenging.

For complex, multi-scale models, a combination of parameter estimation methods is usually employed based on data availability. For example, the earlier discussed model for the self-organisation of hPSC-derived optic-cup organoids [89] obtained some parameters from previous literature and performed specific experiments to measure others (e.g. thickness of the epithelial sheet, length of apical and basal surfaces, and cell density). The free parameters that were not obtained experimentally were constrained through multiple computational simulations to obtain those that recapitulated the optic-cup formation.

The Bayesian paradigm provides a natural mechanism for handling partially observed datasets, incorporating observation errors, and quantifying and propagating the uncertainty in the model parameters and dynamic components. For mechanistic models formed of systems of stochastic differential equations with tractable likelihoods, or suitable tractable approximations to the model likelihood, Markov chain Monte Carlo methods can be used [95, 96], common for applications in systems biology [97, 98]. Such techniques have been employed to infer model parameters for stem cell methylation patterns in colonic crypts, including cell population numbers, niche succession time, and the rate of the methylation/demethylation process [99]. Bayesian inference has also been applied to morphogenesis, treating cells as information processing agents, where the driving force behind morphogenesis is the maximisation of a cell’s model evidence [100]. Furthermore, Bayesian inference techniques have also been used in the development of quantitative measures of corneal transparency [101].

For discrete models, where likelihood-free methods are required, approximate Bayesian computation (ABC) schemes can be employed [102, 103]. Such techniques have been applied to models describing cancer [104] and stem cell division [105] in colonic crypts, quantifying cell-cell adhesion in cell migration in wound healing [106] and stem cell barcoding [107]. Although insightful tools, the implementation of inference schemes can be computationally demanding. This is driving developments in high-performance computing algorithms [108] and applications of supervised ML techniques to accelerate Bayesian inference techniques [109•].

Experimental data are most often described using the properties of a Gaussian random variable (i.e. average values and standard deviations). Although a variety of experimental datasets indeed have Gaussian statistical properties or are closely related (e.g. log-normal), deeper and more sophisticated analyses often involve variables that do not have Gaussian properties. When such variables are measured in a laboratory experiment, their statistical properties and parameters need to be explored and identified and then described in sufficient detail if different experiments are to be compared or if the results are to be used for modelling. Moreover, there are statistical descriptors of spatially distributed signals (such as the distribution of cells of various types in 3D) that cannot be described in terms of specific numerical values. For example, the spatial structures in 3D and their inter-connectedness can be efficiently described in terms of the Betti numbers (see Ref. [110] for an application-oriented review) and represented in the form of a cloud of points in the plane. Such data requires novel statistical criteria to be assessed and compared, and they are an area of active research in statistics [111], including their application to the analysis of diabetic retinopathy images [112•].

## Limitations of Computational Models

Computational methods are driving innovations in diagnostic image analysis and provide a powerful tool for exploring many aspects of hSC behaviour, including the GRN, prediction of cell fate, and self-organisation. However, to ensure the models developed are representative of the real-world system, it is essential that they are underpinned by experimental data. Computational models are designed to complement rather than replace laboratory experiments, with in vitro experimental results driving in silico model development and in silico model results guiding focused in vitro experimentation. Model development also requires non-trivial assumptions to simplify the complex multi-scale physical system into a distilled selection of behaviours of interest, which requires careful navigation using interdisciplinary expertise. A balanced appraisal of the capacities of computational models using examples from embryogenesis is given in Ref. [113].

## Conclusions

The recent advances in the development of state-of-the-art ocular organoids and stem cell therapies hold great promise for translational medical technologies, as well as for deepening our understanding of the fundamental properties of hSC systems and human development. Of key importance to maximising their potential is an interdisciplinary framework that blends sophisticated data analysis and empirical evidence from laboratory experimentation with mathematical and computational modelling.

The stochastic, complex, multi-scale nature of hSC behaviours, particularly when considering 3D organoid structures, makes their quantitative modelling a mathematical and computational challenge, and thus, models often focus on a simplified version of the system or consider a handful of key behaviours to prioritise, based on the research question at hand. It is clear that the most insightful models discussed in this review use computational and in vitro experimentation in a complementary manner.

Advances in ML tools are providing new ways to approach image processing, image classification, in silico model development, and parameter inference. Of particular importance is the potential of ML tools to aid clinical diagnosis and assess response to treatment from IVCM and optical coherence tomography images, as has already been explored for several eye conditions, including LSCD. Future research should expand upon these applications, for example, through the study of IVCM on corneal nerves and dendritic cells in patients with neurotrophic keratopathy [114].

Future computational work will no doubt continue prioritise the application of ML to predicting hSC differentiation pathways and patterning, describing self-organisation behaviours, and enabling diagnostic image analysis and thus will facilitate the development of more comprehensive in silico models of hSCs and computer-aided diagnostic tools.
